# 

**Published:** 1894-11

**Authors:** 


### CONTENTS.

CLINICAL LECTURE—
1. Chronic Gastritis. 2. Pernicious Anemia. 3 Distention of Stomach. By Francis Delafield, M.D., New York ..... 513
ORIGINAL COMMUNICATIONS—
The Medico-Legal, Legal, and Moral Aspects of Criminal Abortion and Infanticide. By Robert Campbell Eve, M.D., Augusta, Ga ..... 516
A Plea for Higher Medical Education in the South. By Wm. Simpson Elkin, M.D., Atlanta, Ga. ..... 531
Fatal Case of Vomiting in Pregnancy, with Notes. By William L. Nolen, M.D., Chattanooga, Tenn ..... 542
SOCIETY REPORTS—
Sixth Annual Session of the Tri-State Medical Society of Alabama, Georgia, and Tennessee, at Atlanta ..... 544
EDITORIAL—
Higher Medical Education ..... 557
Dr. Oliver Wendell Holmes ..... 559
SELECTIONS AND ABSTRACTS—
Eye, Ear, Nose, and Throat ..... 561
General Medicine and Therapeutics ..... 564
MEDICAL ITEMS ..... 571
BOOK REVIEWS ..... 573
ADVERTISERS' INDEX TO ADVERTISEMENTS ..... xiv
CLASSIFIED INDEX TO ADVERTISEMENTS ..... xxxv
ITEMS OF INTEREST ..... x, xi, xii, xiii

